# No evidence for cerebellar abnormality in adults with developmental dyslexia

**DOI:** 10.1007/s00221-018-5351-y

**Published:** 2018-08-16

**Authors:** Casper A. M. M. van Oers, Nadya Goldberg, Gaetano Fiorin, Martijn P. van den Heuvel, L. Jaap Kappelle, Frank N. K. Wijnen

**Affiliations:** 1 Department of Neurology, Brain Center Rudolf Magnus, University Medical Center Utrecht, Utrecht, The Netherlands; 2 Utrecht institute of Linguistics OTS, Utrecht, The Netherlands; 3 Department of Neurology, Amphia Hospital, Breda, The Netherlands

**Keywords:** Dyslexia, MRI, Cerebellum, Voxel-based morphometry

## Abstract

Developmental dyslexia is commonly believed to result from a deficiency in the recognition and processing of speech sounds. According to the cerebellar deficit hypothesis, this phonological deficit is caused by deficient cerebellar function. In the current study, 26 adults with developmental dyslexia and 25 non-dyslexic participants underwent testing of reading-related skills, cerebellar functions, and MRI scanning of the brain. Anatomical assessment of the cerebellum was conducted with voxel-based morphometry. Behavioural evidence, that was indicative of impaired cerebellar function, was found to co-occur with reading impairments in the dyslexic subjects, but a causal relation between the two was not observed. No differences in local grey matter volume, nor in structure–function relationships within the cerebellum were found between the two groups. Possibly, the observed behavioural pattern is due to aberrant white matter connectivity. In conclusion, no support for the cerebellar deficit hypothesis or the presence of anatomical differences of the cerebellum in adults with developmental dyslexia was found.

**Electronic supplementary material:**

The online version of this article (10.1007/s00221-018-5351-y) contains supplementary material, which is available to authorized users.

## Introduction

Developmental dyslexia (DD) is a learning disorder that is characterized by difficulties in accurate and fluent written word recognition. It is associated with poor spelling and reading abilities, despite normal intelligence, normal hearing, adequate classroom exposure, and the absence of physical, emotional or socioeconomic problems (Dickman et al. 2002). DD is regarded as a deficit in the acquisition of written language, which is most likely the result of a deficiency in the recognition and processing of speech sounds (‘phonological deficit’; Boets et al. 2013; Snowling 2000). However, symptoms associated with the condition appear to extend beyond the language domain. Difficulties in motor learning and coordination have been reported in dyslexic individuals. For instance, several studies report that individuals with DD may be less stable during a variety of balancing tasks (Fawcett and Nicolson 1999; Getchell et al. 2007; Moe-Nilssen et al. 2003; Nicolson and Fawcett 1990; Stoodley et al. 2005; Yap and van der Leij 1994), and may be slower in peg moving (Stoodley et al. 2006), rapid pointing (Stoodley et al. 2006a, b; Velay et al. 2002) and bead threading (Nicolson et al. 1994). Implicit perceptual-motor learning, as measured with tasks such as the Serial Reaction Time task (Nissen and Bullemer 1987), is also impaired in people with DD (Howard et al. 2006; Lum et al. 2013; Molinari et al. 1997). In addition, they may show disturbances in binocular control, in tasks that involve reading (Eden et al. 1994; Stein and Fowler 1985) as well as in non-reading processes, such as gaze stability (Bucci et al. 2008a, b; Fischer et al. 2000; Fischer and Hartnegg 2000; Fowler 1991). Furthermore, they may have abnormal control of saccadic eye movements (Biscaldi et al. 1998) and can show deficits in time estimation (Nicolson et al. 1995).

Abovementioned deficiencies in people with dyslexia have led to the cerebellar deficit hypothesis (CDH; Nicolson et al. 1995, 2001). The cerebellum is presumed to regulate processing of information from and to the cortex and other areas by forward loops (Stoodley and Stein 2011). Presumably, the anterior lobe and lobule VII of the cerebellum are involved in fine tuning and supporting processing of sensorimotor programmes, while lobules VI and VII contribute during a wide range of cognitive tasks (Stoodley and Schmahmann 2010). The CDH proposes that deficient phonological processing and awareness, generally assumed to be a proximal cause of difficulty in learning to read, result from impaired articulatory skill acquisition, which in turn results from an ontogenetic cerebellar deficit. Nicolson and Fawcett (2007) incorporated the CDH into Ullman’s (2004) procedural/declarative framework for language functions, and proposed a taxonomy of learning disabilities, based on a division of the procedural learning system into cortico-striatal and cortico-cerebellar subsystems. These investigators argued that the primary neural deficit in dyslexia is caused by functional disturbances in the language-cortico-cerebellar system, although they stated that it is “premature to rule out language-cortico-striatal involvement in dyslexia” (Nicolson and Fawcett 2007). Importantly, both the original CDH and its more recent incarnation are based on cerebellar dysfunction in people with dyslexia. Thus, a critical assumption is that the characteristic symptoms of dyslexia are correlated with the presence of (possibly mild) motor difficulties, and that in the presence of such correlations functional or anatomical abnormalities of the cerebellum should be demonstrable.

The predictions made by the CDH hypothesis have so far not found unequivocal support. The strength of the association between the characteristic literacy-related symptoms of dyslexia and difficulties in motor skill acquisition and motor coordination is still unknown, as is the extent to which such difficulties can be associated with cerebellar abnormalities (Biscaldi et al. 1998; Bucci et al. 2008a, b; Fischer et al. 2000; Fischer and Hartnegg 2000; Getchell et al. 2007; Howard Jr. et al. 2006; Moe-Nilssen et al. 2003; Molinari et al. 1997; Nicolson and Fawcett 1990; Stein and Fowler 1985; Stoodley et al. 2005, 2006a, b; Velay et al. 2002; Yap and van der Leij 1994). In recent meta-analyses of voxel-based morphometry (VBM) studies, grey matter volume reduction in the cerebellum did not reach statistical significance (Eckert et al. 2016; Richlan et al. 2013). However, in a meta-analysis of functional and structural imaging studies, of which four studies were executed in children (5–15 years of age), a reduction of grey matter volume in both hemispheres of the cerebellum in people with DD was observed (Linkersdorfer et al. 2012). A large-scale study in adults showed that local grey matter volume (LGMV) in the right cerebellum (and right lentiform nucleus) of dyslexic people varied considerably, and could be smaller or larger than in the control group (Pernet et al. 2009). In another study with a large sample of adults, spelling was associated with smaller LGMV of the left posterior cerebellum in the control and dyslexic groups combined (Tamboer et al. 2015). Currently, it is unclear whether findings of structural abnormalities in children can be generalized to adults, and whether the language difficulties and motor function impairments observed in dyslexic children persist into adulthood (Ramus et al. 2003a, b). Disappearance of these impairments could imply a developmental delay in maturation of involved brain systems instead of a persisting disorder.

The aim of the present study was to investigate cerebellar involvement in dyslexia in young adults. The questions we addressed were the following: (a) Do young adults with DD show impaired performance on neuropsychological tasks that typically rely on cerebellar function? (b) Are decrements in cerebellar function associated with reduced reading performance? (c) Do adults with DD show deviations in cerebellar structure? (d) Are there any structural deviations in the cerebellum that are associated with reading impairments or (e) with performance on tasks that rely on cerebellar function?

## Methods

### Participants

The dyslexic group included 26 participants (10 males) with a mean age of 23.8 years (SD = 5.3; range 18.7–35.8). The control group included 25 participants (eight males) with a mean age of 23.9 years (SD = 5.6; range 18.4–36.8). Both groups were matched for age and gender (no statistically significant differences). The DD group had higher education than controls (90% versus 81% had more than 12 years education; Mann–Whitney *U* = 418.5, *p* = 0.045).

Dyslexic participants were recruited via advertisements posted at various locations at Utrecht University, the University Medical Center Utrecht, and the University of Applied Sciences Utrecht, as well as via online calls for dyslexic participants on the websites of the Stichting Dyslexie Nederland (Dyslexia Foundation of the Netherlands) and the Faculty of Humanities of Utrecht University, via student counselors at several faculties of Utrecht University and via the ‘Proefbunny online database’ of participants for scientific research. The control participants were selected from the Utrecht Institute of Linguistics OTS participant database as well as from the ‘Proefbunny database’. Written consent was obtained from all participants. The study was approved by the local Medical Ethical Review Board and in accordance with the ethical standards laid down in the 1964 Declaration of Helsinki.

Exclusion criteria were left-handedness (measured by the Edinburgh Lateralization Inventory questionnaire; Oldfield 1971), self-reported hearing or visual impairments, other native language than Dutch, neurological or psychological disorders (in particular attention disorders such as ADD/ADHD), impossibility to enter an MRI scanner, and history of neurological or psychiatric diseases. For participants to be included in the dyslexic group, a formal diagnosis of dyslexia, supplied by an accredited clinical psychologist, was required. The presence of literacy difficulties at the time of participation in the present study was verified with a procedure adopted from Kerkhoff et al. (2013) and Kuijpers et al. (2003). To this end, the one-minute-test (OMT) word reading test (Brus and Voeten 1972) and the ‘Klepel’ pseudo-word reading test (van den Bos et al. 1994), as well as the verbal competence test from the Dutch version of the Wechsler Adult Intelligence Scale (Wechsler 2000) were used. The dyslexic participants needed to meet at least one of the following three criteria to be included: (1) a score below or equal to the 10th percentile on either the OMT or the Klepel test; (2) a score below or equal to the 20th percentile on both the OMT and the Klepel test; or (3) a discrepancy of at least 60 percentile points between the performance on the verbal competence test and the performance on the OMT and the Klepel test. The non-dyslexic participants were not to comply with any of these criteria.

### Neuropsychological test battery

We employed a battery of tests that, in addition to the tests of verbal competence and reading skills mentioned above, comprised tests of automatized lexical access and executive function (Rapid Automatized Naming test), phonological working memory (Non-word Repetition test and Forward and Backward digit span tests), performance IQ (Wechsler Adult Intelligence Scale, Matrix Reasoning) and verbal IQ (Wechsler Adult Intelligence Scale, Vocabulary). The tests were presented in the following order: (1) one-minute test, (2) Klepel test, (3) Non-word repetition test, (4) rapid automatized naming test, (5) WAIS matrix reasoning, (6) WAIS vocabulary, (7) verbal competence test, and (8) digit span forward and backward. Participants also underwent other neuropsychological tests that were not relevant for the purpose of the current study. Participants were tested in a sound-proof cabin at the Utrecht Institute of Linguistics OTS (UiL-OTS). All tests took around 90 min.

The one-minute test (OMT; Brus and Voeten 1972) is a standardized reading test, consisting of a list of 116 semantically unrelated Dutch words printed in four columns on a single sheet. Participants read as many words as possible in 1 min. The number of words the participant reads correctly is tallied.

The Klepel test is a standardized Dutch pseudo-word reading test (van den Bos et al. 1994), which consists of 116 items printed in four columns on a single sheet. Participants read as many pseudo-words as possible within 2 min. The number of words the participant reads correctly was tallied.

The rapid automatized naming test (RAN; Denckla and Rudel 1976) consists of six cards presented in a fixed order, with numbers, capital letters, pictures, lower-case letters, colours, and object icons, respectively. Participants were instructed to name out loud the items (50 per card), as fast and as accurately as possible. The time spent per card was recorded, as well as the number of mistakes. In our analyses, only speed was included.

In the Matrix Reasoning subtest of the Dutch version of the Wechsler Adult Intelligence Scale WAIS_MR, WAIS-III (Wechsler 2000), participants were presented with a matrix in which a piece was missing. They were asked to indicate which item out of five possible alternatives was the missing piece in the pattern. Answers were scored as correct (1) or incorrect (0), with a maximum possible score of 26. In the WAIS-III Vocabulary subtest (WAIS_VC), participants were asked to give a definition of 33 words presented visually and read aloud. Each answer was scored as correct (2), partially correct/incomplete (1) or incorrect (0).

For the verbal competence test (VC), participants were given 20 word pairs (e.g. the pair ‘poem’, ‘statue’) and asked to describe the similarity between the two items of each pair. Answers were scored as correct (2), partially correct/incomplete (1) or incorrect (0).

Working memory was measured with the WAIS-III digit span (DS) subtest (Wechsler 2000) and non-word repetition test (NWRT). We used a Dutch adaptation (de Bree et al. 2007) of the original (Dollaghan and Campbell 1998) non-word repetition test. Participants were instructed to repeat auditorily presented pseudo-words, which complied with phonotactic constraints of Dutch. The number of words the participant repeated correctly was measured. During DS, the experimenter read sequences of digits aloud and the participant was instructed to repeat each sequence as accurately as possible. The sequences were of increasing length. In the second part of the test, the participant was asked to repeat each sequence in reversed order. Two scores were collected corresponding to the number of correctly repeated sequences in the forward and backward conditions.

Cerebellar functions were tested with the bead threading task (BT), a subtest of the Dyslexia screening test (Fawcett and Nicholson 1996) and the time discrimination (TD) task (Nicolson et al. 1995).

In the BT task, participants were provided with a string and 15 wooden beads and were asked to thread the 15 beads as fast as possible, holding the string in the right hand. The relevant measure was the time it took the participant to thread the 15 beads. More brain areas besides the cerebellum are involved during performance of this task, such as the pre- and postcentral gyrus, secondary motor areas and basal ganglia. Nevertheless, the task has been used extensively with the aim to assess cerebellar function in relation to dyslexia (Barth et al. 2010; Irannejad and Savage 2012; Nicolson et al. 1994; Nicolson and Fawcett 1994; Ramus et al. 2003a, b; Savage 2007).

The TD task was used to assess non-motor cerebellar function (Fawcett and Nicholson 1996). Specifically patients with cerebellar lesions as opposed to patients with motor impairment due to different neurological diseases show deficient performance on this task (Ivry and Keele 1989). Better judgment has been associated with higher LGMV in the anterior cerebellum (Hayashi et al. 2014). During the task, participants were presented with pairs of tones, and asked to indicate for each pair whether the second tone was shorter or longer than the first one, by pressing one of two buttons (marked ‘longer’ and ‘shorter’). The first tone in each pair was 1200 ms long with a frequency of 392 Hz. The comparison tones were of longer (1220, 1240, 1260, 1300, 1350, 1400, 1450, 1500, 1600, 1700, or 2000 ms) or shorter duration (1180, 1160, 1140, 1100, 1050, 1000, 950, 900, 850, 800, 700, or 400 ms) but identical in frequency. A 1-s interval separated the first and second stimuli. The task consisted of 66 trials, in which each of the 22 comparison tones was presented three times in a randomized order. A measure of the participant’s ability to distinguish between stimuli that takes a possible response bias into account, *D*’, was calculated as previously described (Stanislaw and Todorov 1999). One control subject and one participant with DD were not tested with TD due to technical issues.

### MRI

Scanning was performed on a 3.0 T Philips Achieva scanner. Anatomical images were acquired with a 3D T_1_-weighted turbo-field echo sequence (flip angle 8°, repetition time = 7.9 ms, echo time = 4.5 ms, inversion time = 955 ms, repetition time of the inversion pulses = 3000 ms, matrix size = 256 × 232, 192 slices, resolution of 1.0 × 1.0 × 1.0 mm^3^). Scan duration was 6 min 43 s.

### Image analysis

For the VBM analysis we used SPM8 (http://www.fil.ion.ucl.ac.uk/spm/). To normalise scans during VBM analysis, typically, a Montreal Neurological Institute (MNI) template is used. However, registration of infra-tentorial structures is known to be less accurate if this template is used, as the larger variability of supra-tentorial structures prohibits precise alignment of the cerebellum. Therefore, we used an SPM toolbox that incorporates a specifically designed spatially unbiased template of the cerebellum and brainstem (SUIT; http://www.diedrichsenlab.org/imaging/suit.htm) to obtain accurate alignment (Diedrichsen et al. 2011).

First, all structural images were manually aligned to the anterior commissure. The cerebellum and brainstem were subsequently isolated using an anatomical mask provided by the toolbox. Resulting images were visually checked and manually corrected if necessary, for example, if occipital grey matter was included. Segmentation was performed subsequently into grey matter, white matter and CSF. Total cerebellar volume was calculated (grey and white matter) and used further down the line to correct for individual cerebellar size. The segmented grey matter images were normalized to the cerebellum (SUIT) template using DARTEL (Ashburner 2007). The images were modulated by the Jacobian determinant, i.e., scaling was performed by the amount of contraction during normalisation. This resulted in sensitivity to differences in volume instead of differences in density. To optimize alignment with the specific atlas, smoothing was performed with a relatively small Gaussian kernel of 6 mm full-width at half maximum (FWHM). Implicit masking was performed to remove voxels of no interest. Finally, images were re-sliced into MNI space for statistical analysis and reporting.

### Statistical analysis

To test group differences in performance on neuropsychological tests, including performance on tests that typically rely on the cerebellum, independent sample *t* tests or Mann–Whitney *U* tests were used, depending on normality of the distribution of variables in each group.

Second, Pearson or Spearman rank correlation, depending on distribution of the outcome variable, was used to assess the association between cerebellar function and reading-related performance in dyslexics, controls and both groups together. Values outside an interquartile range of 1.5 were identified as outliers.

Third, group difference in total cerebellar volume was assessed using a *t* test. For LGMV, a general linearized model, controlling for total cerebellar volume, age and gender, was used.

**Fig. 1:**
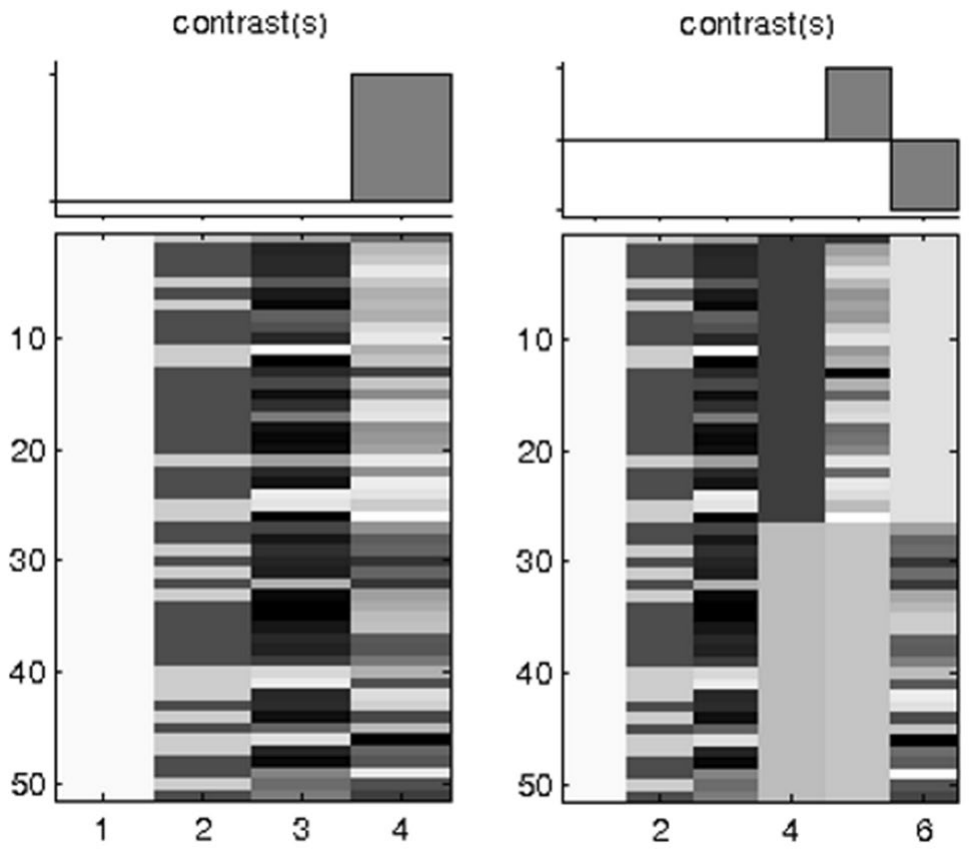
Design matrices for VBM structure–function analysis. Design matrix for structure–function relation; **a** across-group effect with columns for factors: 1, intercept; 2, gender; 3, age; 4, CR; and **b** between-group effect: 1, intercept; 2, gender; 3, group; 4, CR in controls; 5, CR in dyslexics. *CR* composite reading score

Fourth, for assessment of the association between cerebellar morphology and reading-related skills, multiple regression including fixed factors for age, gender was used, while correcting for total cerebellar volume. For all participants, we calculated a composite reading score (CR) to represent ability in literacy-related skills, and, thus, severity of dyslexia. The reasons for doing so were that (1) the individual test scores that made up the CR were highly correlated (Table 1), and (2) it increases statistical power by reducing the number of comparisons. The CR was calculated as the mean of the *z*-scores derived from the OMT, Klepel, NWRT, and RAN scores. Post hoc testing using each individual test score was subsequently conducted. An across-group relation was assessed for CR using multiple regression with age and gender as covariates (Fig. 1a). Subsequently, a multiple regression model with similar covariates as the previous model was created to identify brain areas where dyslexics exhibit a different relationship between CR and Local Grey Matter Volume (LGMV) compared to controls. Additionally, a regressor with group level and a regressor with CR for both groups separately were added (Fig. 1b). This allowed for comparing the structure–function relationships (see below) between both groups. No correction for multiple testing was used to reduce the likelihood of missing relevant results.

**Table 1:** Correlation between components of the composite reading score

	OMT	Klepel test	NWRT
Klepel test	*r* = 0.837***		
NWRT	*r* = 0.464**	*r* =0 .665***	
RAN	*r* = 0.660***	*r* = 0.552***	*r* = 0.304*

*OMT* one-minute test, *NWRT* non-word repetition test, *RAN* rapid automatized naming test

*p < 0.05; **p < 0.01; ***p < 0.001

Fifth, to assess structure–function relationships between cerebellar local grey matter and cerebellar function, similar GLMs were built with BT and TD as parameters.

VBM analyses were corrected at *p* = 0.05 (False discovery rate).

*p* < 0.05 was considered statistically significant.

## Results

### Baseline characteristics, and neuropsychological and literacy tests

Results from neuropsychological tests are shown in Table 2 (baseline characteristics and raw test scores are shown in supplementary Table 1). As expected, participants with dyslexia showed poorer performance on language- and reading-related tests, as well as on tests for short-term and working memory [verbal competence (*p* = 0.038), one-minute-test (*p* < 0.001), Klepel test (*p* < 0.001), non-word repetition (*p* = 0.001), rapid automatized naming (*p* < 0.001), WAIS-III vocabulary (*p* = 0.003), digit span forward (*p* < 0.001) and Digit span backward (*p* = 0.042)], compared to the controls. No significant group differences were found for WAIS-III matrix reasoning.

**Table 2:** Neuropsychological measurements in dyslexics and controls

	Dyslexics (*N* = 26)			Controls (*N* = 25)			Test statistic	*p*
	Mean	Median	SD	Mean	Median	SD		
VC	19.4	19	2.2	20.8	21	2.6	*U* = 216	0.038
OMT	65.8	65	10.0	102.1	104.5	11.6	*t* = 12.2	< 0.001
Klepel test	61.2	64	13.0	105.3	108.5	9.3	*U* = 3.0	< 0.001
NWRT	34.8	33	6.0	40.4	40.5	3.8	*U* = 155.5	0.001
RAN	183.6	186	57.3	130.1	129	21.1	*U* = 559	< 0.001
WAIS-III Matrix Reasoning	21.6	22	2.6	21.4	21	2.6	*U* = 348	0.66
WAIS-III Vocabulary	45.4	46	9.1	51.7	51.5	5.4	*t* = 3.19	0.003
DS forward	8.3	8	1.9	10.5	10	1.8	*t* = 3.93	< 0.001
DS backward	6.5	7	2.0	7.7	7	2.1	*U* = 218.5	0.042
BT	51.9	51	8.0	47.7	47	6.0	*U* = 436.5	0.035
TD	1.82	1.89	0.50	2.12	2.16	0.41	*t* = 2.25	0.029

*VC* verbal competence, *OMT* one-minute test, *NWRT* non-word repetition test, *RAN* rapid automatized naming test, *WAIS-III* Wechsler Adult Intelligence Scale vocabulary subtest, *DS* digit span, *BT* bead threading, *TD* time discrimination

### Cerebellar function tests

**Fig. 2:**
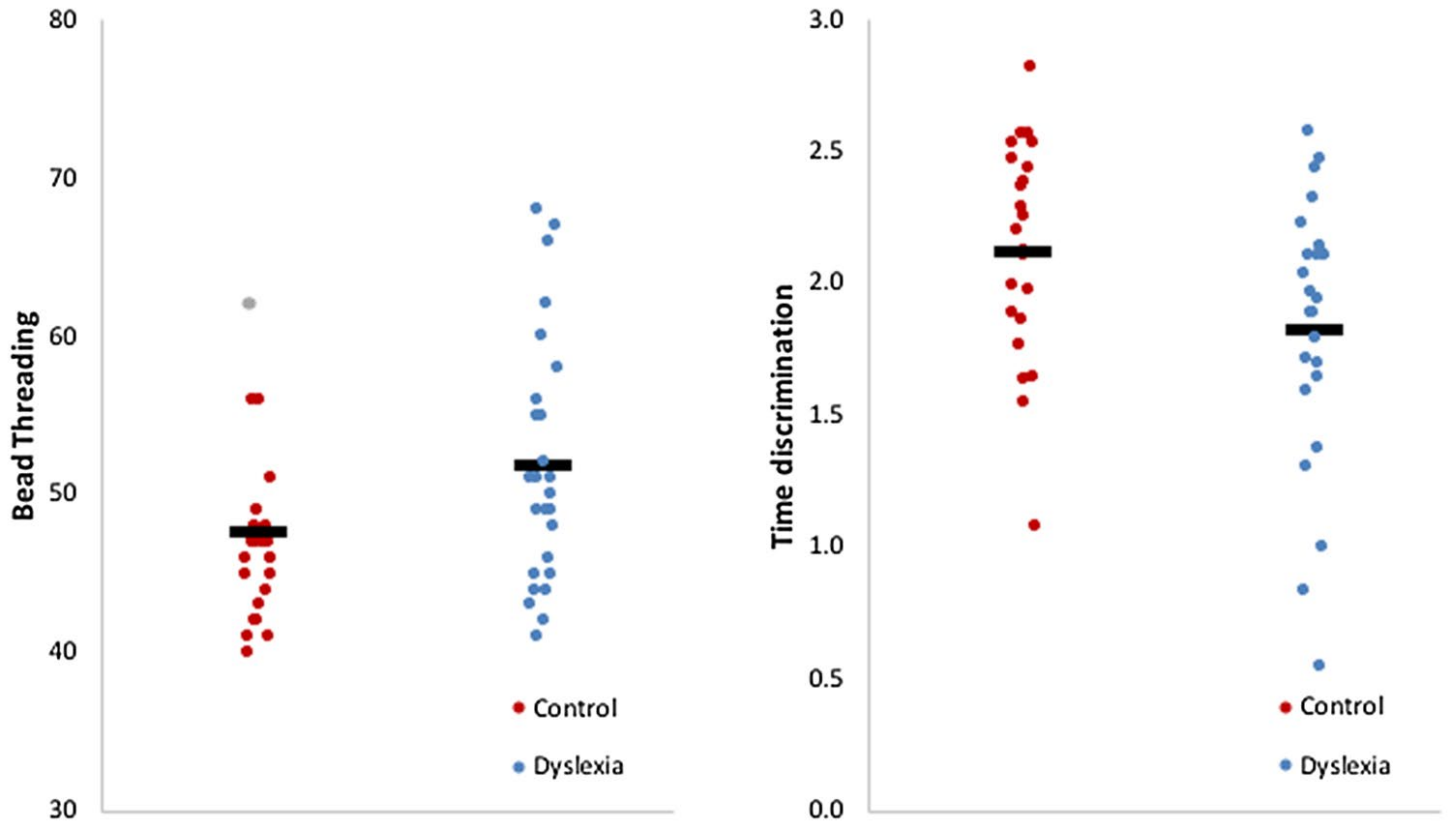
Cerebellar function in dyslexics and controls. Better performance on bead threading (**a**) and time discrimination (**b**) in controls compared to adults with dyslexia. Grey dots represent outliers in the control group for bead threading

Dyslexics showed worse performance on bead threading (BT, 51.8 ± 7.8) than controls (46.4 ± 4.2; *t* = 3.10, *p* = 0.004), as well as on time discrimination (TD, 1.82 ± 0.50) compared to controls (2.12 ± 0.41; *t* = 2.25, *p* = 0.029, Fig. 2). Two outliers were detected and removed from the group comparison and correlation analysis between cerebellar function and literacy skills (see below).

### Relationship between cerebellar function and literacy skills

**Fig. 3:**
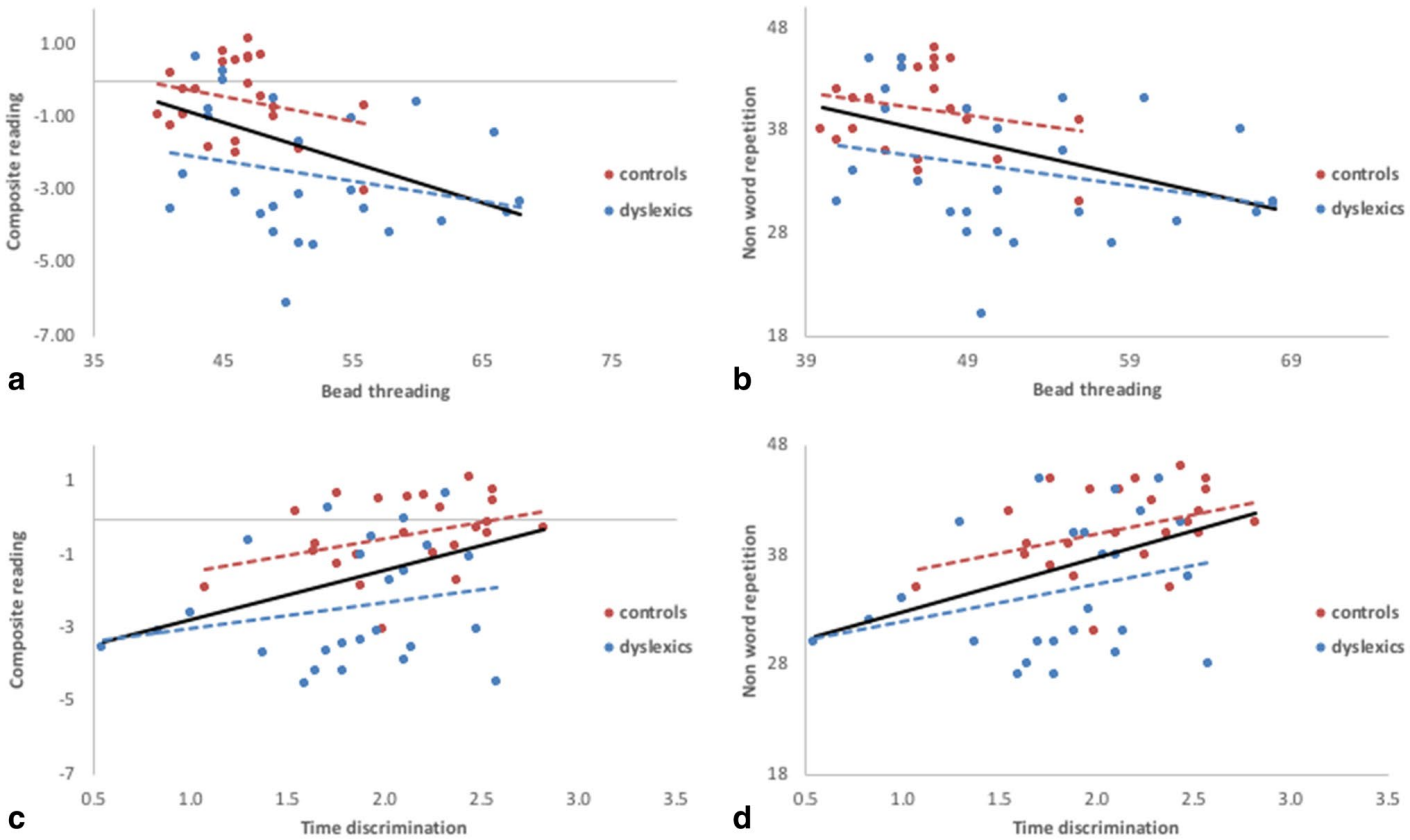
Relation between cerebellar function and literacy skills. Correlation between BT and CR (**a**), BT and NWRT (**b**), TD and CR (**c**), and TD and NWRT (**d**). Solid lines reflect significant correlations across both groups for all plots (**a**–**d**). Dashed lines show non-significant correlations within controls or dyslexics separately

Significant correlations were found between BT and NWRT (*r* = − 0.44; *p* = 0.002), and between BT and the composite reading score (CR, *r* = − 0.46, *p* = 0.001) in the combined dyslexics and control group (Spearman rank tests were used since distributions of outcome variables were not sufficiently close to normal). Besides, TD showed significant correlation with NWRT (*r* = 0.40; *p* = 0.004), RAN (*r* = 0.33, *p* = 0.021), and CR (*r* = 0.39, *p* = 0.006, Fig. 3). These correlations indicated that subjects with low scores on the behavioural tasks informing on cerebellar function (BT, TD) tend to have poorer reading and reading-related performance. However, within the dyslexic or control groups separately, no such correlations were present.

### Differences in cerebellar structure between groups

No group differences in LGMV were observed within the cerebellum, and total cerebellar volume was similar in dyslectics (161.3 ± 18.7 cm^3^) and controls (160.4 ± 14.5 cm^3^, *p* = 0.84).

### Relationship between cerebellar structure and literacy skills and cerebellar function

There were no significant correlations between LGMV and language, reading, and reading-related test scores, nor between LGMV and scores on the behavioural tests associated with cerebellar function (BT, TD), either across both groups or within the dyslexic or control groups.

Post hoc testing of the individual neuropsychological test scores did not yield significant differences.

## Discussion

In this study, using neuropsychological tests and VBM to investigate the role of the cerebellum in DD, dyslexics showed impaired performance on a motor task (BT) and cognitive task (TD) that have previously been associated with cerebellar function. Within the dyslexic group, subjects with worse cerebellar performance did not show larger impairments on literacy skills. In spite of these functional impairments, no differences in LGMV were found in the cerebellum between dyslexics and controls. In addition, no significant correlations were found between volumes of these regions and behavioural measures that represent severity of the reading difficulty or performance of tasks that (partly) rely on the cerebellum.

### Cerebellar function and dyslexia

The individuals diagnosed with dyslexia in this study showed marked deficiency in reading, pseudo-word reading, non-word repetition and rapid automatized naming. At the same time, non-reading skills, such as general intelligence and verbal competence, were unaffected. Together, they make up the core features of the condition and support the validity of the study population.

Several studies have addressed the question if subjects afflicted with DD are impaired in performing motor skills that require cerebellar involvement. Bead threading has been shown to be impaired in children with DD (Nicolson et al. 1994). In our study, on a behavioural level, adult subjects with DD showed signs of impaired cerebellar processing. This is in line with previous data showing that patients with cerebellar lesions show deficient performance on TD as opposed to patients with motor impairments due to damage to other neural circuits (Ivry and Keele 1989). However, we cannot rule out that impaired cortico-striatal processing may also be responsible for our findings.

Ramus et al. (2003) suggested that the occurrence of motor impairments in children with dyslexia results from ADHD or developmental coordination disorder (DCD). On the basis of subjects’ self-reports (requested at inclusion), ADHD or DCD was not prevalent in our study population. Hence, ADHD or DCD co-morbidities are an implausible explanation for our dyslexic participants’ reduced performance on the cerebellar tasks. In a causal relationship, worse cerebellar performance would be expected to be associated with worse literacy skills, which we did not find. This may imply that the relationship between language disorders and cerebellar impairments in the dyslexic group is not causal, but rather the co-occurrence of separate symptoms of the underlying systems disorder, as proposed previously for dyslexic children (Ramus et al. 2003a, b).

### Structure of the cerebellum in dyslexia

Although we found support for the co-existence of a possible cerebellar dysfunction in DD on a behavioural level without strong support for a causal relation with reading skill, no neuroanatomical structural differences were found between participants with DD and healthy controls. Neither did we find a relationship between local grey matter volume and reading skill or cerebellar tasks, even without correction for multiple testing. Therefore, we are confident that no relations between affected language skills and brain structure in our sample of adult with dyslexia were present.

Several studies in DD have investigated differences in brain structure when compared to unimpaired readers using voxel-based morphometry, with or without functional MRI (Brambati et al. 2004; Brown et al. 2001; Dole et al. 2013; Eckert et al. 2005, 2016; Evans et al. 2014; Hoeft et al. 2007; Jednoróg et al. 2014, 2015; Krafnick et al. 2014; Kronbichler et al. 2008; Menghini et al. 2008; Pernet et al. 2009a, b; Raschle et al. 2015; Silani et al. 2005; Siok et al. 2008; Steinbrink et al. 2008; Tamboer et al. 2015; Vinckenbosch et al. 2005; Xia et al. 2016; Yang et al. 2016).

Structural differences in part of the cerebellum were found in five of these studies (Brambati et al. 2004; Brown et al. 2001; Eckert et al. 2005; Kronbichler et al. 2008). Two of these investigated children (Brambati et al. 2004; Brown et al. 2001). In two meta-analyses, structural differences did not reach statistical significance in the cerebellum (Eckert et al. 2016; Richlan et al. 2013). In another meta-analysis of structural and functional imaging studies, a reduction of LGMV in both cerebellar hemispheres was found (Linkersdorfer et al. 2012), four included studies had relatively small sample sizes (10–20), and four studies focused on children. As recently argued by Ramus et al. (2017), sample sizes may have to be much larger than those typically used (10–20) to have an acceptable chance of detecting a difference. Our study is in line with the absence of clear consistent differences in LGMV in the cerebellum found in previous studies. A study by Pernet et al. (2009) re-analyzing an earlier sample, used a probabilistic atlas created from typical readers matched to the dyslexic ones. They assessed if each voxel in the dyslexics was outside of the norm in the control group. In the right hemisphere of the cerebellum, all dyslexics differed from the control group by exhibiting either increased or decreased local brain volume. This finding may suggest a more complex and possibly non-linear relationship between grey matter volume and functional properties of the network. Besides, dyslexia may consist of different subgroups that exhibit different functional–anatomical signatures. These effects may not be visible in larger samples with larger heterogeneity. Possibly, the absence of a significant effect in our study may be related to this issue. In the future, characterization of subgroups of DD is critical in studies with large sample sizes.

If DD is a developmental disorder of brain networks involved in the acquisition of reading skills, the anatomical signature of DD may be very strong in young children but may diminish at a later age. Indeed in children, growing experience with reading can result in increase of local grey matter volume in several brain areas including the right cerebellum (Krafnick et al. 2011). It has, therefore, been postulated that observed differences in brain structure or function may be a consequence of long-term reduction of exposure to reading or an altered cognitive strategy, rather than the neurobiological cause of the reading deficit (Norton et al. 2015). If affected brain structures recover later in adulthood, it would explain the absence of a difference found in our study.

Rather than macroscopic abnormalities, the basis for deficient literacy-related skills in dyslexia may be reflected by microstructural changes. Abnormal processing within grey matter of the cerebellum may, therefore, still be present, potentially caused by deviant intracerebellar connectivity. Indeed, functional imaging studies have shown altered cerebellar activation in response to relevant cognitive tasks (for review see Linkersdorfer et al. 2012).

Besides, altered long-range connectivity with cortical areas through brainstem and thalamic nuclei could be responsible for abnormal cortico-cerebellar processing and consequent diminished reading skills (Balsters et al. 2013; Müller-Axt et al. 2017). Disruption of multiple supra-tentorial white matter tracts of the classical reading network, and connections to and from the cerebellum have been observed in dyslexia (Cui et al. 2016; Fernandez et al. 2016; Sotero and Trujillo-Barreto 2007). Moreover, white matter tract differences in the left hemisphere may already be observed in pre-readers at risk for dyslexia (Vanderauwera et al. 2017; Vandermosten et al. 2012). Increase of fractional anisotropy (FA), reflective of white matter integrity, of the right superior longitudinal fasciculus (which includes the arcuate) in dyslexic children, has been found to be associated with improvement of reading skills (Hoeft et al. 2011). Candidate genes for dyslexia and specific language impairment have been shown to be related to white matter structure (Marino et al. 2014; Scerri et al. 2012). Nevertheless, a recent meta-analysis did not find consistent differences in local FA between subjects with and without dyslexia (Moreau et al. 2018). Further studies on white matter connectivity between the cerebellum and the cerebral cortex are needed, in which challenges related to small sample sizes, differences in methods and individual anatomical heterogeneity need to be addressed.

The genetic background of DD strongly implies dysfunctional neuronal migration and neurite outgrowth (for review Poelmans et al. 2011). Although genetic influence may determine literacy outcome by modulation of plasticity of specific critical brain areas (Männel et al. 2015; Skeide et al. 2016), it seems plausible that a more widespread dysfunction of grey and white matter networks may be responsible. This could explain the structural cerebellar changes found in previous studies, in the absence of a (clear) direct causal link between cerebellar function and reading impairments.

## Conclusions

In our study, adults with developmental dyslexia showed impaired performance on a motor task and cognitive task that rely on the cerebellum, but we found no clear support for a causal relation between cerebellar function and reading skills. This finding, together with corresponding results of earlier studies, suggests that hypotheses that put much weight on persistent cerebellar dysfunction as a crucial factor in the pathogenesis of dyslexia (CDHs) may need to be reconsidered.

The co-occurrence of cerebellar dysfunction and dyslexia could be related to involvement of more widespread neural circuitry. Since we did not find morphological changes of the cerebellum, co-occurrence of cerebellar impairment in subjects with developmental dyslexia may be mediated by widespread microstructural changes or deviant white matter connectivity at large-scale network level.

## Electronic supplementary material

Below is the link to the electronic supplementary material.

Supplementary material 1 (DOCX 18 KB)
