# Supplementary material for: No evidence for cerebellar abnormality in adults with developmental dyslexia
Source: Exp Brain Res. 2018 Aug 16;236(11):2991–3001. doi: 10.1007/s00221-018-5351-y (PMC6223834; doi:10.1007/s00221-018-5351-y)
Supplement: Supplementary file 1 — Supplementary material 1 (DOCX 18 KB) [file 221_2018_5351_MOESM1_ESM.docx]

**Supplementary Table 1**

Baseline characteristics and raw test scores

| subject | age (yrs) | gender | Education | Composite reading (z) | VC | OMT | Klepel | NWRT | RAN | WAIS VC | WAIS MR | DS FW | DS BW | BT | TD |
| --- | --- | --- | --- | --- | --- | --- | --- | --- | --- | --- | --- | --- | --- | --- | --- |
| C1 | 19,1 | male | 1 | -0,69 | 21 | 105 | 109 | 39 | 135 | 45 | 20 | 10 | 5 | 56 | 1,64 |
| C2 | 21,3 | female | 1 | 0,65 | 20 | 101 | 94 | 45 | 140 | 53 | 24 | 12 | 7 | 47 | 2,20 |
| C3 | 21,4 | female | 1 | 0,58 | 22 | 115 | 113 | 44 | 105 | 58 | 24 | 14 | 12 | 47 | 2,12 |
| C4 | 20,3 | female | 1 | -1,00 | 23 | 87 | 86 | 39 | 160 | 52 | 24 | 10 | 7 | 49 | 1,86 |
| C5 | 21,3 | female | 1 | -0,25 | 22 | 110 | 102 | 41 | 123 | 57 | 20 | 8 | 9 | 42 | 2,82 |
| C6 | 24,0 | female | 1 | -0,43 | 17 | 106 | 111 | 40 | 130 | 53 | 20 | 11 | 6 | 48 | 2,53 |
| C7 | 19,7 | female | 1 | 0,76 | 24 | 105 | 112 | 45 | 119 | 55 | 21 | 12 | 8 | 45 | 2,57 |
| C8 | 21,2 | female | 1 | -0,75 | 23 | 93 | 91 | 40 | 136 | 49 | 21 | 10 | 7 | 49 | 2,36 |
| C9 | 18,4 | male | 1 | 1,14 | 20 | 115 | 114 | 46 | 116 | 51 | 24 | 11 | 8 | 47 | 2,44 |
| C10 | 19,5 | female | 2 | -1,26 | 17 | 100 | 109 | 37 | 132 | 47 | 18 | 8 | 6 | 41 | 1,76 |
| C11 | 25,6 | female | 2 | -0,94 | 23 | 114 | 110 | 38 | 111 | 49 | 15 | 9 | 8 | 42 | 1,64 |
| C12 | 25,1 | male | 2 | -0,27 | 21 | 115 | 108 | 41 | 95 | 45 | 24 | 9 | 11 | 43 | 2,47 |
| C13 | 21,9 | female | 2 | 0,28 | 20 | 107 | 108 | 43 | 126 | 46 | 22 | 13 | 5 | 62 | 2,29 |
| C14 | 19,8 | female | 1 | -1,89 | 19 | 100 | 103 | 35 | 117 | 41 | 22 | 9 | 5 | 51 | 1,08 |
| C15 | 18,8 | male | 1 | -0,41 | 19 | 104 | 111 | 40 | 140 | 57 | 21 | 9 | 6 | 62 | 2,10 |
| C16 | 19,0 | female | 1 | -1,69 | 13 | 116 | 107 | 35 | 111 | 44 | 16 | 11 | 6 | 46 | 2,38 |
| C17 | 35,3 | male | 1 | -0,12 | 20 | 90 | 111 | 42 | 130 | 56 | 21 | 11 | 10 | 47 | 2,53 |
| C18 | 35,8 | female | 1 | 0,50 | 22 | 98 | 113 | 44 | 131 | 56 | 21 | 12 | 7 | 45 | 2,57 |
| C19 | 30,4 | male | 2 | 0,68 | 25 | 93 | 112 | 45 | 131 | 61 | 19 | 10 | 10 | 48 | 1,76 |
| C20 | 36,8 | male | 1 | -0,94 | 23 | 109 | 107 | 38 | 128 | 59 | 25 | 9 | 8 | 40 | 2,25 |
| C21 | 24,5 | female | 1 | 0,19 | 22 | 116 | 114 | 42 | 125 | 50 | 24 | 11 | 9 | 41 | 1,55 |
| C22 | 21,3 | female | 1 | -1,87 | 22 | 98 | 79 | 36 | 122 | 49 | 21 | 9 | 5 | 44 | 1,89 |
| C23 | 27,8 | female | 1 | 0,55 | 20 | 81 | 105 | 44 | 201 | 57 | 24 | 15 | 12 | 46 | 1,97 |
| C24 | 19,3 | female | 4 | -1,98 | 17 | 98 | 111 | 34 | 140 | 53 | 21 | 6 | 6 | 46 |  |
| C25 | 30,2 | male | 2 | -3,05 | 20 | 73 | 99 | 31 | 159 | 50 | 23 | 10 | 7 | 56 | 1,99 |
| D1 | 35,0 | male | 3 | -6,12 | 20 | 59 | 15 | 20 | 294 | 38 | 23 | 5 | 3 | 50 |  |
| D2 | 19,2 | female | 4 | -3,56 | 24 | 63 | 58 | 31 | 158 | 35 | 22 | 9 | 5 | 41 | 2,14 |
| D3 | 26,0 | female | 1 | -0,78 | 18 | 74 | 64 | 42 | 124 | 54 | 22 | 9 | 7 | 44 | 2,23 |
| D4 | 34,3 | male | 2 | -1,01 | 19 | 83 | 74 | 40 | 140 | 51 | 20 | 8 | 4 | 44 | 1,89 |
| D5 | 20,8 | female | 1 | -3,53 | 20 | 77 | 65 | 30 | 169 | 46 | 22 | 7 | 4 | 56 | 0,55 |
| D6 | 21,4 | male | 1 | 0,26 | 18 | 66 | 73 | 45 | 189 | 49 | 25 | 10 | 6 | 45 | 1,71 |
| D7 | 27,6 | female | 2 | -4,20 | 18 | 52 | 53 | 28 | 220 | 36 | 22 | 6 | 7 | 49 | 1,64 |
| D8 | 20,0 | female | 1 | -0,52 | 21 | 64 | 77 | 40 | 285 | 54 | 18 | 10 | 11 | 49 | 1,94 |
| D9 | 19,0 | male | 2 | -1,46 | 21 | 73 | 68 | 38 | 186 | 55 | 24 | 12 | 11 | 66 | 2,10 |
| D10 | 28,3 | female | 1 | 0,66 | 20 | 59 | 80 | 45 | 277 | 52 | 23 | 11 | 9 | 43 | 2,32 |
| D11 | 23,6 | female | 1 | -1,71 | 23 | 57 | 64 | 38 | 183 | 59 | 25 | 8 | 6 | 51 | 2,03 |
| D12 | 21,5 | female | 1 | -3,47 | 17 | 73 | 66 | 30 | 190 | 47 | 16 | 8 | 7 | 49 | 1,79 |
| D13 | 21,5 | female | 1 | -4,20 | 16 | 53 | 62 | 27 | 251 | 55 | 21 | 8 | 5 | 58 | 1,79 |
| D14 | 21,0 | male | 2 | -3,06 | 19 | 56 | 59 | 36 | 13 | 39 | 22 | 9 | 7 | 55 | 2,47 |
| D15 | 18,7 | female | 2 | -0,60 | 19 | 59 | 90 | 41 | 201 | 42 | 16 | 8 | 7 | 60 | 1,30 |
| D16 | 19,3 | male | 1 | -3,91 | 21 | 75 | 54 | 29 | 168 | 56 | 20 | 7 | 5 | 62 | 2,10 |
| D17 | 18,8 | female | 1 | -1,04 | 21 | 70 | 64 | 41 | 133 | 48 | 21 | 12 | 7 | 55 | 2,44 |
| D18 | 26,6 | male | 1 | -3,70 | 18 | 75 | 66 | 30 | 134 | 41 | 23 | 10 | 7 | 48 | 1,38 |
| D19 | 21,8 | female | 2 | -0,01 | 18 | 51 | 48 | 44 | 273 | 40 | 26 | 8 | 9 | 45 | 2,10 |
| D20 | 21,2 | female | 6 | -4,54 | 20 | 55 | 49 | 27 | 200 | 24 | 18 | 7 | 4 | 52 | 1,59 |
| D21 | 22,0 | male | 1 | -3,09 | 21 | 58 | 39 | 33 | 203 | 45 | 22 | 6 | 8 | 46 | 1,97 |
| D22 | 31,5 | female | 3 | -4,47 | 17 | 58 | 31 | 28 | 190 | 30 | 24 | 6 | 5 | 51 | 2,58 |
| D23 | 19,9 | female | 3 | -3,35 | 16 | 70 | 47 | 31 | 208 | 35 | 20 | 8 | 4 | 68 | 1,89 |
| D24 | 35,8 | male | 2 | -3,65 | 19 | 87 | 62 | 30 | 124 | 57 | 23 | 9 | 7 | 67 | 1,70 |
| D25 | 23,2 | female | 2 | -2,59 | 23 | 65 | 65 | 34 | 186 | 44 | 22 | 8 | 6 | 42 | 1,00 |
| D26 | 20,5 | male | 2 | -3,12 | 17 | 73 | 52 | 32 | 186 | 42 | 23 | 4 | 4 | 51 | 0,84 |

Subjects: C control, D dyslexic; Education: 1 University master degree, 2 higher vocational education, 3 intermediate vocational education, 4 higher secondary school, 5 intermediate secondary school, 6 lower secondary school; VC verbal competence; OMT one-minute test; NWRT non-word repetition test; RAN rapid automatized naming test; WAIS VC Wechsler Adult Intelligence Scale vocabulary subtest; WAIS MR Wechsler Adult Intelligence Scale matrix reasoning subtest; DS FW digit span forward; DS BW digit span backward; BT bead threading; TD time discrimination
